# The postpartum depression literacy scale (PoDLiS): development and psychometric properties

**DOI:** 10.1186/s12884-019-2705-9

**Published:** 2020-01-03

**Authors:** Fatemeh Mirsalimi, Fazlollah Ghofranipour, Azita Noroozi, Ali Montazeri

**Affiliations:** 1 Department of Health Education and Promotion, Faculty of Medical Sciences, Tarbiat Modares University, Tehran, Iran; 2 Department of Health, Bushehr University of Medical Sciences, Bushehr, Iran; 3 Population Health Research Group, Health Metrics Research Center, Iranian Institute for Health Sciences Research, ACECR, Tehran, Iran

**Keywords:** The postpartum depression literacy scale, Psychometric properties, Perinatal women

## Abstract

**Background:**

Maternal mental health including postpartum mental health is essential to women’s health. This study aimed to develop a specific measure for assessing postpartum depression literacy and consequently evaluate its psychometric properties among a sample of perinatal women.

**Methods:**

This investigation was composed of two studies: developing the measure, and evaluating of psychometric properties of the developed questionnaire. In development stage an item pool was created. Then, based on definition of mental health literacy and preliminary screening, an initial questionnaire was developed. The content and face validity of the questionnaire were then assessed. In the second study psychometric properties of the questionnaire were examined. Overall 692 perinatal women with the mean age of 27.63 years (ranging from 17 to 43) participated in the study.

**Results:**

In all an item pool of 86 items was generated. Of these, 31 items were removed and the remaining 55 items subjected to content and face validity and further 16 items removed. In the second stage a 39-item questionnaire namely the Postpartum Depression Literacy Scale (PoDLis) was evaluated. In principal component factor analysis, 31 items were loaded indicating a 7-factor solution for the questionnaire. The factors designated the following constructs: ability to recognize postpartum depression, knowledge of risk factors and causes, knowledge and belief of self-care activities, knowledge about professional help available, beliefs about professional help available, attitudes which facilitate recognition of postpartum depression and appropriate help-seeking, and knowledge of how to seek information related to postpartum depression. Finally performing the confirmatory factor analysis, the Postpartum Depression Literacy Scale with 31 items was supported for the structures suggested by theoretical model and findings from the exploratory factor analysis. The Cronbach’s alpha coefficient for the scale was .78 and it ranged from .70 to .83 for each factor lending support to the internal consistency of the questionnaire.

**Conclusion:**

The findings suggest that the Postpartum Depression Literacy Scale (PoDLiS) is a reliable and valid instrument for measuring the postpartum depression literacy and now can be used in studies of mental health literacy in women.

## Background

Postpartum depression is a common problem among women [1, 2]. Postpartum depression can negatively impact the health and well-being of the mother and babies. Untreated postpartum depression can extend the probability of chronic mental health and suicidal behaviors. Furthermore, the insecure attachments are more likely to be developed between infants and depressed mothers. Also it has been reported that infants of such as mothers may have more developmental and behavioral problems [3].

Only about 40% of women with depression are identified by health care providers and a significant percentage of women do not obtain treatment for their depressive symptoms [4]. Also, usually most women do not proactively seek professional help for signs and symptoms of depression during the postpartum period, even if treatment is offered and accessible [5–7]. The lack of knowledge about signs and symptoms of depression and treatment possibilities, have been considered as a major help-seeking barrier during the postpartum period [5, 6], indicating how significant is the role of women’s depression literacy in the help-seeking process [8]. Thus providing the knowledge and skills are essential for women to recognize postpartum depression and obtain efficient treatment [9]. Postpartum depression literacy may be conceived as a particular type of mental health literacy, defined as the knowledge and beliefs about mental health disorders that aid their recognition, management or prevention [8].

Mental health literacy consists of the following attributes: (a) the ability to recognize mental health disorders, (b) knowledge and beliefs about risk factors and causes, (c) knowledge and beliefs regarding self-help strategies, (d) knowledge and beliefs of professional help and treatment options, (e) attitudes which promote recognition and appropriate help-seeking, and (f) knowledge of how to seek mental health information [10].

However, currently no instrument exists for measuring postpartum depression literacy within mental health literacy framework. To the best of our knowledge, there are only two studies that measured postpartum depression literacy but none of them used a specific measure: Thorsteinsson et al. in their study, applied three vignettes that met the diagnostic requirements for major depressive episode as per the fifth edition of the Diagnostic and Statistical Manual of Mental Disorders (DSM-V) [11]. Fonceca et al. in a study used the Portuguese version of the depression literacy questionnaire [12] which consisted of 22 true/false/do not know items. Each correct answer was scored 1, and the total score ranged from 0 to 22 points. The Cronbach’s alpha coefficient for the questionnaire (to assess internal consistency) was .77 [13].

However, as one might consider there is a gap in the literature and therefore, we tried to overcome this shortcoming by developing an instrument for measuring postpartum depression literacy in women in order to facilitate future studies on the topic with a broader application. In particular, the present study aimed to develop a specific scale of mental health literacy for postpartum depression and then provide an evaluation of its psychometric properties. We anticipated that this new scale might be used both as a screening measure of literacy and as a measure for assessing the impact of interventions on the promotion of mental health literacy among women.

## Methods

### Study 1: Developing the measure

Study 1 included four stages of item generation, content validity, face validity and providing provisional version of the questionnaire.

#### Item generation

A review of all measures of mental health literacy and qualitative studies was carried out in order to generate an item pool. As such a number of items were adapted from other measures [11, 12, 14–27]. Then, based on the Jorm’s definition of mental health literacy [8], an initial questionnaire was developed. The initial questionnaire contained 86 items. After a careful review of the items by the research team, the number of items was reduced to 55. Operational definitions of the six attributes of mental health literacy and decisions regarding item generation are presented in the Table 1.

**Table 1:** The operational definitions for the Postpartum Depression Literacy Scale (PoDLiS) attributes and rationale for item generation

Attributes of the mental health literacy	Attributes of the postpartum depression literacy	Development rationale	Number of items
the ability to recognize mental health disorders	The ability to recognize postpartum depression	Items were adapted based on: - The Depression Literacy Questionnaire (D-Lit) [12] (e.g., sleeping too much or too little may be a sign of depression, eating too much or losing interest in food may be a sign of depression, depression affects person’s memory and concentration and people with depression often hear voices that are not there), - The Mental Health Literacy questionnaire (MHLq) in young people [14] (e.g., one of the symptoms of depression is the loss of interest or pleasure in most things), - The Vignette Interview developed by Jorm in 2010 [11] and - An item based on Diagnostic and Statistical Manual of Mental Disorders V TR criteria in 2013 [15] (e.g., symptoms and signs of postpartum depression last for a period of at least 2 weeks).	7
Knowledge and beliefs about risk factors and causes	Knowledge of risk factors and causes	Items were adapted or generated based on: -The MacArthur Mental Health Module [16] (e.g., in your opinion, how likely is it that NAME’s situation might be caused by a genetic or inherited problem?, in your opinion, how likely is it that NAME’s situation might be caused by stressful circumstances in his/her life? and in your opinion, how likely is it that NAME’s situation might be caused by bad character?), - The work of Thorsteinsson et al. in 2014 [11] (e.g., hormonal changes, lack of social support, financial problems and obstetric factors), - The qualitative study of Ugarriza in 2002 [17] (e.g., difficult or unsuccessful breastfeeding and inability to have a vaginal delivery) and -The study of Robertson et al. in 2004 [18] (e.g., a previous history of depression).	10
knowledge and beliefs regarding self-help strategies	Knowledge and beliefs of self-care activities	Items included knowledge and beliefs of common strategies typically recommended such as physical activity, good sleep and having a balanced diet. Items were adapted or generated based on: - The qualitative study of Guy et al. in 2014 [19] (e.g., getting out of the house, seeking employment, allowing emotions out through activities like crying and going to church), -The work of Thorsteinsson et al. in 2014 [11] (e.g., childcare), - The qualitative study of Abrams et al. in 2009 [20] (e.g., religious practices, prayer and physical exercise), -The work of Letourneau et al. in 2007 [21] and Ugarriza in 2002 [17] (e.g., infant care and household chores from intimate partners, trusted family members, and friends), and - The Mental Health Literacy questionnaire (MHLq) in young people [14] (e.g., good sleep helps to improve mental health, having a balanced diet helps to improve mental health).	9
knowledge and beliefs of professional help and treatment options	Knowledge and beliefs about professional help available	Items included knowledge and beliefs of mental health professionals and the services they provide. Items were included or adapted based on: - The Depression Literacy Questionnaire (D-Lit) [12] (e.g., clinical psychologists can prescribe antidepressants, most people with depression need to be hospitalized, people with depression should stop taking antidepressants as soon as they feel better and antidepressants are addictive). - The scale of attitudes toward seeking psychological help [22] (e.g., although there are clinics for people with mental troubles, I would not have much faith in them and a person with an emotional problem is not likely to solve it alone, he is likely to solve it with professional help), - The Mental Health Knowledge Schedule (MAKS) [23] [e.g., psychotherapy (for example, talking therapy or counselling) can be an effective treatment for people with mental health problems], - The study of Angermeyer et al. in 1993 [24] (e.g., If taken for long, these drugs cause irreversible brain damage), and -The mental health literacy scale (MHLS) [25] (e.g., I believe treatment for a mental illness, provided by a mental health professional, would not be effective).	9
Attitudes which promote recognition and appropriate help-seeking	Attitudes which facilitate recognition of postpartum depression and appropriate help-seeking	Items included attitudes that impact on recognition of postpartum depression and willingness to engage in help-seeking behavior. Items were adapted or included based on: - The mental health literacy scale (MHLS) [25] (e.g., people with a mental illness could snap out of it if they wanted, a mental illness is a sign of personal weakness, It is best to avoid people with a mental illness so that you don’t develop this problem, If I had a mental illness I would not tell anyone, If I had a mental illness, I would not seek help from a mental health professional and seeing a mental health professional means you are not strong enough to manage your own difficulties), - The Barriers Scale [7] (e.g., be afraid of what my family and/or friends might think of me for attending psychology and/or psychiatry appointments), - The study of Mcluckie et al. in 2014 [26] (e.g., Most people who have a mental illness are dangerous and violent), - The scale of attitudes toward seeking psychological help [22] (e.g., I would rather live with certain mental conflicts than go through the ordeal of getting psychiatric treatment and emotional difficulties, like many things, tend to work out by themselves) and - The study of Abrams et al. in 2009 [20] (e.g., good mothers don’t get depressed, People tell me this is normal and PPD means you’re crazy).	13
Knowledge of how to seek mental health information	Knowledge of how to seek information related to postpartum depression	Items were adapted based on: - The mental health literacy scale (MHLS) [25] [e.g., I am confident that I know where to seek information about mental illness, I am confident using the computer or telephone to seek information about mental illness and I am confident I have access to resources (e.g., GP, internet, friends) that I can use to seek information about mental illness], - The health literacy measure for adolescents (HELMA) [27] (e.g., I am able to ask others about health information that I need). - Items were also included appraisal of information and generated by authors (e.g., I can appraise the accuracy of information about postpartum depression on the radio and television, I can appraise the accuracy of information about postpartum depression on the Internet and I can appraise the accuracy of advices about postpartum depression which given me by friends and family members).	7

Note. Response format for all items was a 5-point Likert type scale ranging from 1 to 5 (1 = strongly disagree or not likely at all and 5 = strongly agree or very likely; reverse items score oppositely)

#### Content validity

Both qualitative and quantitative content analysis was performed. For qualitative content validity 15 experts in health education and promotion, reproductive health and psychology were requested to assess the questionnaire for grammar, wording, item allocation, and scaling. After collecting experts’ opinions, essential changes were considered in the questionnaire and 11 items were removed. For quantitative content validity, in order to calculate the Content Validity Ratio (CVR), the same experts were asked to assess each item on a 3-point Likert scale (where 1 = essential, 2 = useful but unessential, and 3 = unessential). Then, based on Lawshe’s table [28], the values greater than or equal to 0.49 were kept in the scale. In this stage, five items were removed. In order to calculate the Content Validity Index (CVI), the experts were asked to determine the relevance, clarity and simplicity of items using a 4-point Likert scale. A CVI score of 0.79 or above indicates good content validity [29]. The CVI score for all questions was calculated and found to be satisfactory (ranging from 0.80 to 1). Content validity ratio; and content validity index were made simultaneously.

#### Face validity

Similar to previous stage both qualitative and quantitative methods were used. The qualitative face validity was carried out by using open-ended questions. As such 15 perinatal women were recruited using convenience sampling to determine the ambiguity, relevance and difficulty of each item. At this stage, none of the items were deleted. At the quantitative stage, the impact score of each item was calculated. For this purpose, another sample of 40 perinatal women was requested to identify the importance of each item on a 5-point Likert scale including very important, important, relatively important, slightly important, and unimportant. Then impact score was calculated. For calculation of item impact score, first the percentage of women who scored 4 to 5 for each item (frequency) was indicated, and then item impact score of instrument items was calculated by following formula: Item impact score = frequency (%) × importance. The items with impact scores of 1.5 or above was considered acceptable [30, 31]. The impact score for items ranged from 1.8 to 3.8.

#### Provisional version of the questionnaire

Following the content and face validity, the provisional version of the questionnaire with 39 items was provided and subjected to psychometric evaluation. The questionnaire was named the Postpartum Depression Literacy Scale (PoDLiS). Each item is rated on a 5-point Likert scale ranging from 1 to 5 (1 = strongly disagree or not likely at all and 5 = strongly agree or very likely, reverse items score oppositely). Scores for each subscale and the whole questionnaire determined by summing items dividing into the number of items for each subscale and for the whole questionnaire giving a score of 1 to 5 either for each subscale or the whole questionnaire.

### Study 2: Psychometric properties of the postpartum depression literacy scale

#### Design and participants

A cross-sectional study was conducted on a convenient sample of pregnant and postnatal women attending to the prenatal and pediatric clinics of a teaching hospital affiliated to Tehran University of Medical Sciences. Eligibility criteria to take part in the study were as follows: being 18 years or older, being currently pregnant or having given birth during the previous 12 months, and having ability to read and write properly (had to have completed primary education). As suggested we estimated that a sample of 400 perinatal women would be enough for this study (10 individuals per item of the questionnaire) [32]. However, in practice 447 women participated in the study and completed the PoDLiS. In addition since we performed confirmatory factory analysis a different sample (*n* = 245) was recruited. In fact overall 692 perinatal women participated in the study.

#### Statistical analysis

Construct validity and reliability were performed to assess psychometric properties of the questionnaire. To investigate the construct validity, exploratory and confirmatory factor analyses were used. To assess reliability, internal consistency analysis was performed. These are described as follows.

#### Exploratory factor analysis (EFA)

The principal component analysis was conducted on 39 items using oblique rotation. The oblique methods allow the factors to correlate if we believe that the factors (latent concepts) are correlated [33]. To determine the number of potential underlying factors, eigenvalues above 1, a minimum factor loading of 0.4, a maximum of 25 rotation iterations and a scree plot were used [34, 35]. Also the rotation of factor analysis converged at six iterations. To assess the appropriateness of the sample for the factor analysis, The Kaiser-Meyer-Olkin (KMO) and Bartlett’s Test of Sphericity were applied [35, 36].

#### Confirmatory factor analysis (CFA)

The confirmatory factor analysis was conducted to test whether the data fit theoretical model. The CFA analysis carried out according to the Jorm’s model (6 attributes) and the findings from the exploratory factor analysis using covariance indexes and the maximum-likelihood estimation method. The goodness-of-fit of the model was assessed using relative chi-square (χ^2^/df), the goodness-of-fit index (GFI), the comparative fit index (CFI), the normed fit index (NFI), the root mean square error of approximation (RMSEA), and the standardized root mean square residual (SRMR) [37]. For goodness-of-fit indexes, the following values thought acceptable: were χ^2^/df ≤ 2, GFI, CFI, and NFI > .95, RMSEA < .06, and SRMR < .08 [38, 39].

#### Reliability

The Cronbach’s alpha coefficient was used to assess internal consistency of the whole scale and each factor separately.

## Results

### The sample characteristics

The mean age of respondents was 27.63 (SD = 5.46) years and it was 12.99 (SD = 2.46) years for their formal education. Almost 90% of the participants were housewife and 42.1% of respondents said that psychologists were their first source of seeking help if they would suffer from postpartum depression. The characteristics of the study participants are presented in Table 2.

**Table 2:** Socio-demographic and clinical characteristics of the sample of perinatal women

Measure	*M* (SD) */ n* (%)
Age (years)	27.63 (5.46)
Education (years)	12.99 (2.46)
Occupational status	
Housewife	625 (90.3)
Employed	40 (5.8)
Student	27 (3.9)
Spouse education	11.90 (3.12)
Spouse job	
Employed	665 (96.2)
Unemployed	27 (3.8)
Household economic status	
Good	216 (31.2)
Neither good nor bad	450 (65.0)
Bad	21 (3.0)
Very bad	5 (0.8)
Perinatal period	
Pregnancy	609 (88.0)
Postpartum	83 (12.0)
Parity	
Primiparity	471 (68.1)
Multiparity	221 (31.9)
Source of seeking help about postpartum depression	
General practitioner	23 (3.3)
Obstetrician	73 (10.5)
Psychiatrist	71 (10.3)
Psychologist	291 (42.1)
Midwife	15 (2.2)
Friends, family members	188 (27.2)
Source of seeking information about postpartum depression	
General practitioner	103 (14.9)
Obstetrician	73 (10.5)
Psychiatrist	34 (4.9)
Psychologist	98 (14.2)
Midwife	17 (2.5)
Friends, family members	36 (5.2)
Internet	188 (27.2)
Book	33 (4.8)
Radio and television	21 (3.0)
I don’t know where to get the information	82 (11.8)

### Exploratory factor structure

After confirming the adequacy of the sampling based on the KMO and Bartlett’s Test of Sphericity (KMO = .783) and χ^2^ (741, *n* = 447) = 4449, *P* < .001 (two-tailed), 11 factors emerged with eigenvalues of greater than 1, which accounted for 60.63% of the variance observed. By using the scree plot [40] and according to the dimensions of mental health literacy, the number of factors was limited to 7 with loading 31 items and an explanation for 49% of the total variance. These seven factors are presented as follows: ability to recognize postpartum depression (6 items), knowledge of risk factors and causes (5 items), knowledge and belief of self-care activities (5 items), knowledge about professional help available (2 items), beliefs about professional help available (2 items), attitudes that facilitate recognition of postpartum depression and appropriate help-seeking (6 items) and knowledge of how to seek information related to postpartum depression (5 items). The results are shown in Table 3.

**Table 3:** Factor Loadings for Exploratory Factor Analysis with oblique rotation of the Postpartum Depression Literacy Scale (PoDLiS)

	Item	A1	A2	A3	A4	A5	A6	A7
1	Feeling unusually sad and teary may be a symptom of postpartum depression	**.61**	.00	.12	.14	−.06	.11	−.40
2	Sleeping too much or too little may be a sign of postpartum depression	**.76**	−.02	.15	.18	−.01	.08	−.21
3	Eating too much or losing interest in food may be a sign of postpartum depression	**.74**	−.13	.17	.11	.09	.14	−.21
4	Loss of interest or pleasure in most things may be a symptom of postpartum depression	**.64**	.02	.18	.18	−.05	.28	−.44
5	Postpartum depression affects person’s memory and concentration	**.53**	.08	.11	.11	−.04	.32	−.41
6	Symptoms and signs of postpartum depression last for a period of at least 2 weeks	**.59**	−.06	.22	.20	.05	.38	−.28
7	How likely is it that postpartum depression might be caused by a genetic or inherited problem	.38	−.23	.09	.13	.09	.07	**−.52**
8	How likely is it that postpartum depression might be caused by stressful circumstances in the life (such as the death of a loved one or divorce)?	.18	−.00	.19	.22	.07	.26	**−.81**
9	How likely is it that postpartum depression might be caused by lock of social support such as intimate partner support?	.25	.21	.13	.19	.01	.20	**−.81**
10	How likely is it that postpartum depression might be caused by a previous history of depression?	.32	−.04	.16	.06	−.07	.17	**−.77**
11	How likely is it that postpartum depression might be caused by a hormonal imbalance?	.47	.05	.18	.19	.03	.16	**−.53**
12	Physical activity is effective for the prevention or management of postpartum depression	.12	.02	**.63**	.09	−.03	.28	−.23
13	Seeking help with tasks like infant care and house hold chores from intimate partners and family members is helpful for the prevention or management of postpartum depression	.08	.02	**.69**	.16	−.14	.18	−.21
14	Religious practices, prayer and going to holy shrine are helpful for the prevention or management of postpartum depression	.08	−.02	**.76**	.11	−.04	.17	−.09
15	Having a balanced diet is helpful for the prevention or management of postpartum depression	.15	−.12	**.78**	.07	.07	.10	−.03
16	Good sleep is helpful in prevention or management of postpartum depression	.21	−.06	**.79**	.16	.03	.11	−.11
18	Although there are clinics for with postpartum depression, I would not have much faith in them	.07	**.52**	.06	−.01	.26	.25	−.12
22	Treatment for postpartum depression, provided by a mental health professional, can be effective	.12	−.04	.19	.15	−.10	**.90**	−.14
23	Psychotherapy (for example, talking therapy or counselling) can be effective in treating postpartum depression	.14	−.00	.17	.18	−.07	**.90**	−.20
24	Antidepressants are addictive	−.03	.10	−.04	−.03	**.88**	−.11	.01
25	Antidepressants cause brain damage	.03	.26	−.06	−.10	**.81**	−.05	−.02
26	I would rather live with postpartum depression than go through the ordeal of getting psychiatric treatment	−.04	**.61**	.05	−.12	.11	.13	−.00
30	Most women who have postpartum depression are violent	−.11	**.59**	−.16	−.15	.06	−.27	.11
31	It is best to avoid women with postpartum depression so that you don’t develop this problem	−.04	**.77**	−.04	−.07	.17	−.02	−.14
32	If I had postpartum depression I would not tell anyone	.02	**.59**	−.01	−.09	.28	.13	.08
34	I am afraid of what my family and/or friends might think of me for attending psychology and/or psychiatry appointments	.17	**.41**	−.03	−.07	.12	−.01	.03
35	I know where to seek information about postpartum depression	.16	−.14	.18	**.56**	−.05	.18	−.16
36	I know how to use various sources to seek information about postpartum depression	.20	.04	.13	**.66**	.07	.15	−.16
37	I can appraise the accuracy of information about postpartum depression on the radio and television	.12	−.18	.11	**.76**	.06	.14	−.16
38	I can appraise the accuracy of information about postpartum depression on the Internet	.07	−.01	.11	**.78**	−.08	.11	−.15
39	I can appraise the accuracy of advices about postpartum depression which given me by friends and family members	.12	−.14	.12	**.68**	−.21	.13	−.07
Eigenvalue		6.01	3.65	2.49	1.99	1.84	1.66	1.47
Explained Variance (%)		15.43	9.36	6.39	5.10	4.71	4.26	3.77

Note. Factor loadings ≥ .40 are in boldface. A = Attribute. A1 = Ability to recognize postpartum depression; A2 = Attitudes which facilitate recognition of postpartum depression and appropriate help seeking; A3: Knowledge and beliefs of self-care activities; A4: Knowledge of how to seek information related to postpartum depression; A5: Beliefs about professional help available; A6: Knowledge about professional help available; A7: Knowledge of risk factors and causes

### Confirmatory factor structure

**Fig. 1:**
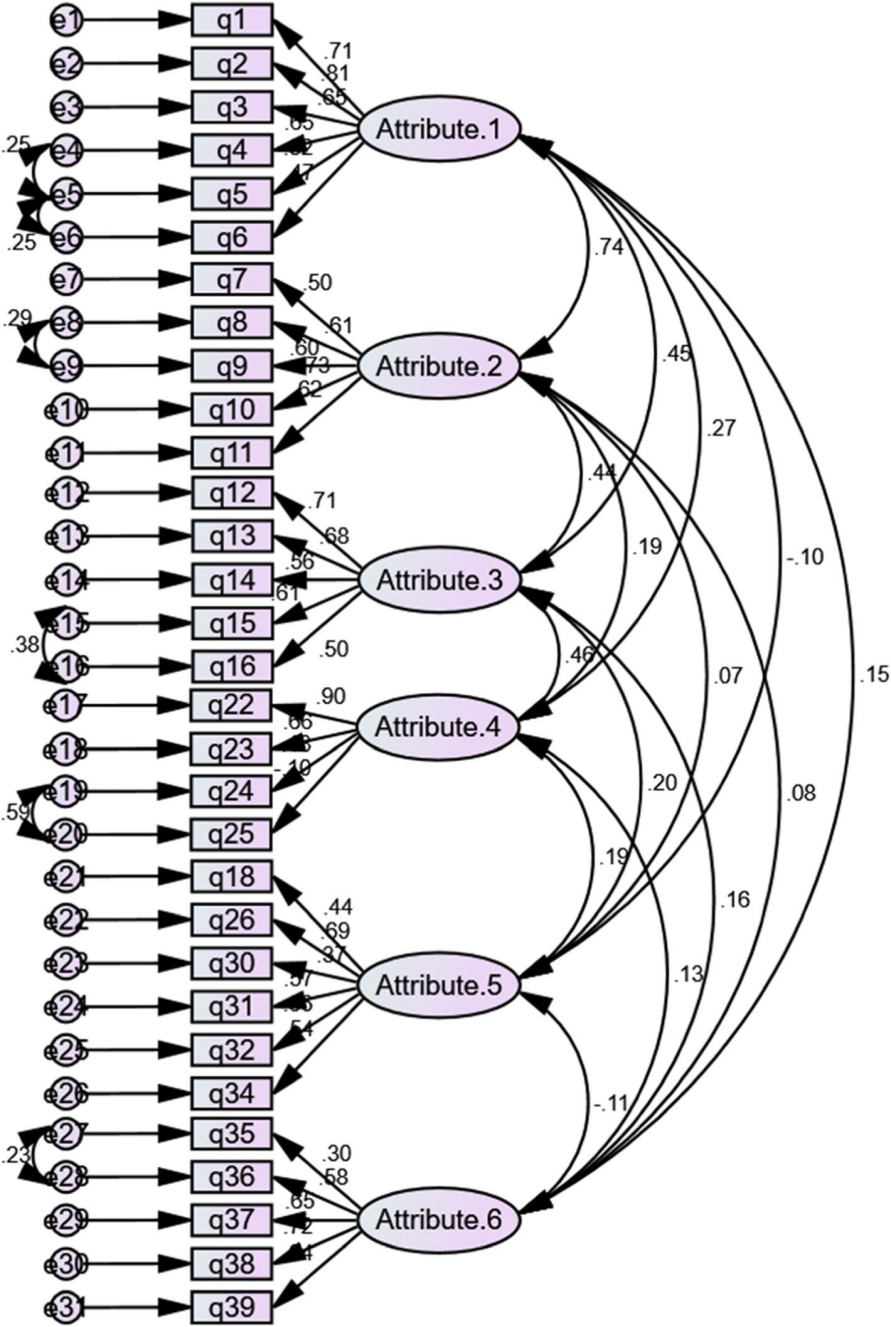
The result obtained from confirmatory factor analysis for the Jorm’s model

**Fig. 2:**
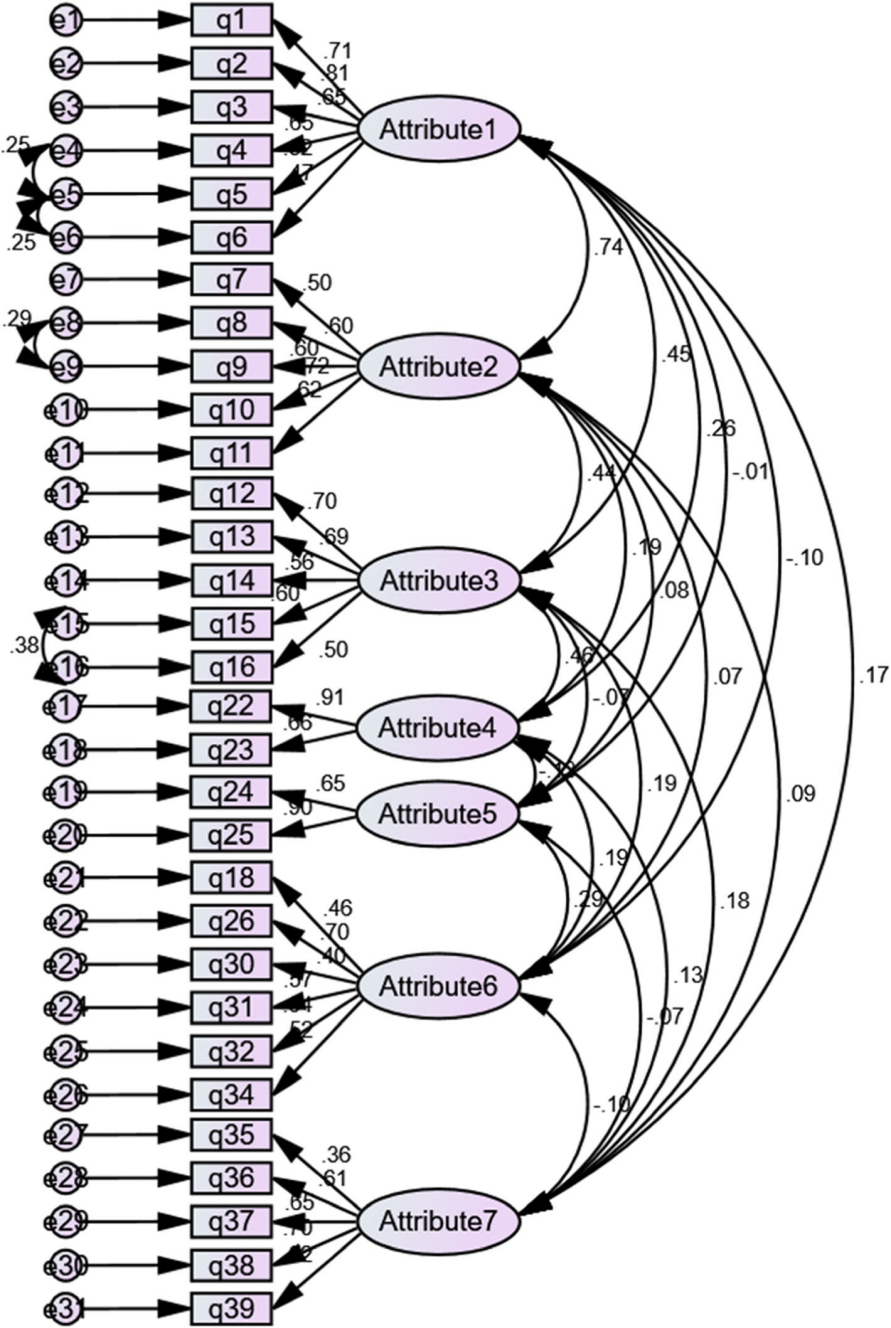
The result obtained from confirmatory factor analysis for the PoDLiS

The CFA was performed on six attributes of mental health literacy according to the Jorm’s model to check the theoretical model fit. The results showed a good fit to the data. The goodness fit indexes for the proposed model were: root mean square error of approximation (RMSEA) = .040, normal fit index (NFI) = .764, comparative fit index (CFI) = .919, incremental fit index (IFI) = .921, goodness-of-fit index (GFI) = .871, and standardized root mean square residual (SRMR) = .074 (Table 4). The results of the confirmatory factor analysis are shown in Fig. 1. However, as shown in Table 4 we performed the CAF analysis for the seven attributes of the instrument derived from the exploratory factor analysis and found that this model also revealed a good fit to the data with a very small better goodness of fit indexes (Fig. 2).

**Table 4:** The fit indicators of the Jorm’s model and the Postpartum Depression Literacy Scale (PoDLiS)

Model	Indicator level
Jorm’s model (six attributes)	
Chi-squared *P* value (χ^2^)	< .001
χ^2^/df	1.38
RMSEA	.040
NFI	.764
CFI	.919
IFI	.921
GFI	.871
SRMR	.074
PoDLiS model (seven attributes)	
Chi-squared *P* value (χ^2^)	< .001
χ^2^/df	1.38
RMSEA	.039
NFI	.767
CFI	.921
IFI	.923
GFI	.872
SRMR	.067

### Reliability

The Cornbrash’s alpha coefficient for the Postpartum Depression Literacy Scale was .78 and for ability to recognize postpartum depression was .77 and for risk factors and causes, knowledge and beliefs of self-care activities, knowledge about professional help available, beliefs about professional help available, attitudes which facilitate recognition of postpartum depression and appropriate help-seeking and knowledge of how to seek information related to postpartum depression were .76, 78, .83, .78, .70, .73, respectively. The results are shown in Table 5.

**Table 5:** Cronbach’s α coefficient for the Postpartum Depression Literacy Scale (PoDLiS) and its attributes

Attributes	Number of items	*M* (*SD*)	Possible range	Cronbach’s α coefficient (*n* = 692)
Ability to recognize postpartum depression	6	3.68 (0.75)	1–5	.77
Knowledge of risk factors and causes	5	3.69 (0.77)	1–5	.76
Knowledge and beliefs of self-care activities	5	4.50 (0.56)	1–5	.78
Knowledge about professional help available	2	4.10 (0.91)	1–5	.83
Beliefs about professional help available	2	2.53 (1.08)	1–5	.78
Attitudes which facilitate recognition of postpartum depression and appropriate help-seeking	6	3.77 (0.78)	1–5	.70
Knowledge of how to seek information related to postpartum depression	5	3.71 (0.77)	1–5	.73
Total scale	31	3.79 (0.39)	1–5	.78

### Postpartum depression literacy score

The mean postpartum depression literacy for the study sample was found to be 3.79 (SD =0.39) and it was 3.68 (SD = 0.75) for ability to recognize postpartum depression, 3.69 (SD = 0.77), 4.50 (SD = 0.56), 4.10 (SD = 0.91), 2.53 (SD = 1.08), 3.77 (SD = 0.78) and 3.71 (SD = 0.77) were for knowledge of risk factors and causes, knowledge and beliefs of self-care activities, knowledge about professional help available, beliefs about professional help available, attitudes which facilitate recognition of postpartum depression and appropriate help-seeking, knowledge of how to seek information related to postpartum depression respectively.

### The relationship between postpartum depression literacy and socio-demographic and clinical characteristics

There were statistically significant differences in the postpartum depression literacy score by age, education, occupation and source of seeking information about postpartum depression. Univariate analyses showed that older age, higher education and being employed and student were associated with higher postpartum depression literacy. Also, respondents who said book, the Internet, psychologists, psychiatrics, radio and television were their first source of seeking information had higher postpartum depression literacy (Table 6).

**Table 6:** The relationship between characteristics of the participants and the postpartum depression literacy

Variables	*n*	*M* (*SD*)	t/f	P
Age			−1.97	.049٭
< 30	440	3.77 (0.38)		
≥ 30	252	3.83 (0.39)		
Education			**−6.02**	**< .001٭**
≤ 12	452	3.73 (0.38)		
> 12	240	3.91 (0.36)		
Occupational status			**7.17**	**.001٭**
Housewife	625	3.77 (0.38)		
Employed	40	3.95 (0.41)		
Student	27	3.97 (0.28)		
Household economic status			**.77**	**.511**
Good	216	3.81		
Neither good nor bad	450	3.79		
Bad	21	3.71		
Very bad	5	3.63		
Perinatal period			**−1.54**	**.122**
Pregnancy	609	3.78 (0.39)		
Postpartum	83	3.85		
Source of seeking information about postpartum depression			**4.54**	**< .001٭٭**
General practitioner	103	3.71 (0.37)		
Obstetrician	73	3.74 (0.41)		
Psychiatrist	34	3.87 (0.33)		
Psychologist	98	3.89 (0.37)		
Midwife	17	3.57 (0.40)		
Friends, family members	36	3.67 (0.43)		
Internet	188	3.86 (0.35)		
Book	33	3.94 (0.46)		
Radio and television	21	3.86 (0.32)		
I don’t know where to get the information	82	3.68 (0.36)		
Parity			**.82**	**.408**
Primiparity	471	3.80 (0.39)		
Multiparity	221	3.77 (0.37)		

٭*P* < .05 ٭٭ *P* < .001

## Discussion

This study was the first attempt to design and psychometrically evaluate an instrument to measure postpartum depression literacy among women. The initial questionnaire was developed based on review of all measures of mental health literacy, qualitative studies and definition of mental health literacy and its frameworks [41]. After the completion of the validity and reliability phases, the Postpartum Depression Literacy Scale (PoDLiS) consisted of 31 items tapping into 7 factors that now can be used to evaluate knowledge of signs and symptoms of postpartum depression and attitudes toward mental health and help-seeking. One of the features of the PoDLiS is that, in addition to assessment of each component of the mental health literacy as relates to postpartum depression, existence of scoring system included a coalition of all attributes of mental health literacy and provided a theoretically meaningful structure for the scale. As most participants completed the questionnaire without any problems in about 15 min, we believe that the PoDLiS is an easy-to-use questionnaire and can be utilized in future studies.

The current study used both exploratory and confirmatory factor analyses to assess the structure of the PoDLiS with almost 692 perinatal women and the EFA revealed seven attributes factorial structure for the scale. This however was a slightly different from the framework that indicated by Jorm. However, the confirmatory factor analysis revealed a satisfactory fit between the data and both six attributes of the original Jorm’s model and the seven attributes that we extracted from the exploratory factor analysis. The Jorm’s framework contains six attributes (the ability to recognize mental health disorders, knowledge and beliefs about risk factors and causes, knowledge and beliefs regarding self-help strategies, knowledge and beliefs of professional help and treatment options, attitudes which promote recognition and appropriate help-seeking and knowledge of how to seek mental health information) while as mentioned earlier in EFA analysis we found seven factors for the PoDLiS. In fact knowledge and beliefs about professional help available in our study was divided into two constructs: knowledge about professional help seeking and beliefs about professional help seeking.

According to the results, 42.1% of respondents said that psychologists were their first source of seeking help if they would suffer from postpartum depression following by friends and family members in second place. Also, 27.2% of respondents said that the Internet was their first source of seeking information following by psychologists in second place. The results of an Australian study found that general physicians were the primary source of seeking help and information for the presented vignette [11].

The findings revealed that the mean score of more attributes of postpartum depression literacy such as ability to recognize postpartum depression, knowledge of risk factors and causes, attitudes which promote recognition of postpartum depression and appropriate help-seeking and knowledge of how to seek information related to postpartum depression were these all in the moderate range. Thus, the need to improve knowledge about postpartum depression symptoms, risk factors, causes and treatment by educational interventions among women during the perinatal period seems essential. Also, specific strategies should be developed in order to overcome postpartum depression stigma.

Most women stated that the Internet was the first source of seeking information about postpartum depression. Indeed conducting interventions to assess the effects of credible online information on women’s postpartum depression literacy might be helpful.

Furthermore, our results showed that the highest and lowest mean scores of different dimensions were knowledge and beliefs on self-care activities and beliefs about professional help available respectively. Health professionals should provide women with education during pregnancy about self-care activities such as physical activity that may lessen distress during the postpartum period [42]. Teaching about the use of antidepressants as a treatment option should be a priority for health professionals.

Participants in our study had good knowledge of mental health professionals and the services they provide. It may be helpful to provide information about professional help and treatment options, as it will help women make right decisions about their mental health.

We showed that overall women presented moderate level of postpartum depression literacy during the perinatal period, which was consistent with the results of Fonceca et al. [13]. This finding showed that women at perinatal period did not have adequate knowledge about postpartum depression, suggesting the need for exploring and developing facilitators such as postpartum depression literacy that affecting women’s help-seeking behaviors. Education should be provided through community campaigns, as well as by presenting mental health in childbirth classes during pregnancy [43, 44].

Finally, we explored the relationship between sociodemographic and clinical variables and the postpartum depression literacy. Differences were found regarding age, education, occupation and source of information about postpartum depression. Fonceca et al. showed that among sociodemographic variables, education and income levels correlated with the depression literacy and based on clinical variables, there was not any correlation between perinatal period and parity with the depression literacy [13]. These results were in line with those seen in our study.

### Limitations

This study has some limitations. First, this was a hospital-based study. Thus the sample might not be a representative of perinatal population. Future studies might benefit from including a community sample of women. Secondly, intention to develop a brief and easily administered measure may have resulted in insufficient assessment of its attributes. However, multiple sources were used to achieve consensus in guiding item development and testing, including use of the literature, the research team (experts) and a broad testing process. Finally, we did not perform convergent and discriminant validity and test-retest reliability. The future studies should include more psychometric analysis, such as convergent and discriminant validity and test-retest reliability.

### Implications and future research

Use of The Postpartum Depression Literacy Scale will enable efficient identification of high risk women who may benefit from further education or support. This scale may also allow the detection of changes within an individual or population in order to assess the impact of programs to improve postpartum depression literacy and prevent or manage postpartum depression.

In addition future studies should be carried out in different environments and cultures. Perhaps the evaluation of such studies may lead to a stronger confirmation of the psychometric properties of the PoDLiS. However, responsiveness of the scale (sensitivity to change) will be assessed by researchers following this study.

## Conclusion

The Postpartum Depression Literacy Scale provided a valid measure to assess all attributes of mental health literacy related to postpartum depression and an understanding of knowledge and beliefs about postpartum depression among perinatal women. This understanding is necessary to improving mental health status of women. It has good psychometric properties and easily administered. The Postpartum Depression Literacy Scale (PoDLiS) can be used in assessing level of postpartum depression literacy in pregnant women or new mothers and in determining the impact of programs designed to improve mental health literacy related to postpartum depression.

## Data Availability

The datasets generated and analyzed during the current study are not publicly available due to restriction by Tarbiat Modares University but are available from the corresponding author on reasonable request.

## Abbreviations

CFA: Confirmatory factor analysis
CFI: Comparative fit index
CVI: Content validity Index
CVR: Content validity Ratio
DSM-V: Diagnostic and Statistical Manual of Mental Disorders fifth edition
EFA: Exploratory factor analysis
GFI: Goodness-of-fit index
IFI: Incremental fit index
KMO: Kaiser-Meyer-Olkin
NFI: Normal fit index
PoDLiS: Postpartum Depression Literacy Scale
RMSEA: Root mean square error of approximation
SRMR: Standardized root mean square residual
